# Mechanical Stretching Simulates Cardiac Physiology and Pathology through Mechanosensor Piezo1

**DOI:** 10.3390/jcm7110410

**Published:** 2018-11-02

**Authors:** Tzyy-Yue Wong, Wang-Chuan Juang, Chia-Ti Tsai, Ching-Jiunn Tseng, Wen-Hsien Lee, Sheng-Nan Chang, Pei-Wen Cheng

**Affiliations:** 1 Research Assistant Center, Show Chwan Memorial Hospital, Changhua 50000, Taiwan; wongtzyyyue@gmail.com; 2 Department of Emergency, Kaohsiung Veterans General Hospital, Kaohsiung 81300, Taiwan; wcchuang@vghks.gov.tw; 3 Department of Business Management, National Sun Yat-Sen University, Kaohsiung 80424, Taiwan; 4 Division of Cardiology, Department of Internal Medicine, National Taiwan University Hospital, Taipei 10048, Taiwan; cttsai1999@gmail.com; 5 Department of Education and Research, Kaohsiung Veterans General Hospital, Kaohsiung 81300, Taiwan; cjtseng@vghks.gov.tw; 6 Graduate Institute of Clinical Medicine, College of Medicine, Kaohsiung Medical University, Kaohsiung 81700, Taiwan; cooky-kmu@yahoo.com.tw; 7 Department of Internal Medicine, Kaohsiung Municipal Hsiao-Kang Hospital, Kaohsiung Medical University, Kaohsiung 81700, Taiwan; 8 Department of Internal Medicine, School of Medicine, College of Medicine, Kaohsiung Medical University, Kaohsiung 81700, Taiwan; 9 Graduate Institute of Clinical Medicine, National Taiwan University College of Medicine, Taipei 10051, Taiwan; 10 Division of Cardiology, Department of Internal Medicine, National Taiwan University Hospital Yun-Lin Branch, Dou-Liu City 64069, Taiwan; 11 Yuh-Ing Junior College of Health Care & Management, Kaohsiung 82100, Taiwan; 12 Shu-Zen Junior College of Medicine and Management, Kaohsiung 80700, Taiwan

**Keywords:** mechanical stimulation, Piezo1, cardiomyocytes, stretching

## Abstract

The dynamics of a living body enables organs to experience mechanical stimulation at cellular level. The human cardiomyocytes cell line provides a source for simulating heart dynamics; however, a limited understanding of the mechanical stimulation effect on them has restricted potential applications. Here, we investigated the effect of mechanical stimulation on the cardiac function-associated protein expressions in human cardiomyocytes. Human cardiomyocyte cell line AC16 was subjected to different stresses: 5% mild and 25% aggressive, at 1 Hz for 24 h. The stretched cardiomyocytes showed down-regulated Piezo1, phosphorylated-Ak transforming serine473 (P-AKT^S473^), and phosphorylated-glycogen synthase kinase-3 beta serine9 P-GSK3β^S9^ compared to no stretch. In addition, the stretched cardiomyocytes showed increased low-density lipoprotein receptor-related protein 6 (LRP6), and phosphorylated-c-Jun N-terminal kinase threonine183/tyrosine185 (P-JNK^T183/Y185^). When Piezo inhibitor was added to the cells, the LRP6, and P-JNK^T183/Y185^ were further increased under 25%, but not 5%, suggesting that higher mechanical stress further activated the wingless integrated-(Wnt)-related signaling pathway when Piezo1 was inhibited. Supporting this idea, when Piezo1 was inhibited, the expression of phosphorylated-endothelial nitric oxide synthase serine1177 (P-eNOS^S1177^) and release of calcium ions were reduced under 25% compared to 5%. These studies demonstrate that cyclic mechanical stimulation affects cardiac function-associated protein expressions, and Piezo1 plays a role in the protein regulation.

## 1. Introduction

The advancement for whole heart modelling is required for understanding cardiac dynamics; however, developing a whole heart model is difficult. As a result, mechanical stimulation has recently been applied to study cell dynamics in vitro. The goal of mechanical stimulation technology is to develop models that mimic in vivo dynamics. The hypertensive heart induces heart failure due to mechanical overloading greater than 140 mmHg [1]. Mechanical forces such as stretching, bending, and compression regulate cardiac structure and function [2]. The cardiac cells express mechano-sensing proteins such as integrins and Angiotensin II Type 1 Receptors (AT1R) in the cell membrane [3]. The mechanically activated proteins may have roles in cardiac overloading; however, it has not been well studied in cardiac cells. 

The limitations in cardiac cell model have been the lack of cardiac cells that beat spontaneously when cultured in vitro. In vivo study has shown that cardiac overloading is a form of excessive mechanical stimulation, and mechanical stretching can mimic cardiac hypertrophy with increased protein expressions related to oxidative stress such as c-Jun N-terminal kinase JNK [4], and nitric oxide (NO) [5]. The cardiovascular cells and cardiomyocytes express endothelial nitric oxide synthetase (eNOS) [6] whose function can be modulated by mechanically activated ion channels such as Piezo [7,8]. Emerging evidence has shown that β-adrenergic receptor (β-AR) compensated for the dysfunctional eNOS protein during cardiac hypertrophy [9]. Overexpression of eNOS promotes survival rate of congestive heart failure patient; however, patients showed decreased cardiac contractile function [10]. Cardiac cell contractility is stimulated by norepinephrine to activate protein kinase A (PKA) through β-adrenergic receptor which in turn phosphorylates sarcolemma reticulum (SR) calcium channels, and increases intracellular calcium release [11,12]. The cardiac cells with decreased contractility survived cardiac overloading through unknown mechanisms. We hypothesized that the mechanically activated protein Piezo1 plays a role in regulating eNOS expression in the overloaded cardiac cells. In this study, we investigated the impact of mild and aggressive cyclic stretching on human cardiomyocytes. Discerning the underlying mechanism on how cardiomyocytes sense the mechanical cue in microenvironment is of great importance for developing accurate in vitro cardiac models. 

## 2. Materials and Methods

### 2.1. Cell Culture

Adult human ventricular cardiomyocyte 16 AC16 were a gift from Professor Po-Lin Kuo in the Kaohsiung Medical University. The cells were maintained in DMEM/F12 (GeneDireX Incorporation, Taoyuan, Taiwan), supplemented with penicillin and streptomycin (Thermo Fisher Scientific, Waltham, MA, USA), and 12.5% fetal bovine serum (Fisher Scientific, Pittsburgh, PA, USA). The cells were incubated in humidified atmosphere at 37 °C, with 5% CO_2_. The cells were passaged every 3–4 days, passages 2 to 10 were used in this study. 

### 2.2. Stretching Device

The stretching device ARTEMIS ATMS Boxer was sponsored by the manufacturer TAIHOYA Corporation (Kaohsiung, Taiwan). The ATMS Boxer was placed in the CO_2_ incubator at 37 °C during stretching. The stretching device is composed of chambers which accommodate cells cultured on the polydimethylsiloxane (PDMS). The stretchable PDMS membranes were purchased from Genemessenger (Kaohsiung, Taiwan). The PDMS surface area for cell adhesion was 2 × 2 cm^2^, or 2 × 5 cm^2^. The cells were seeded on membrane pre-coated with Collagen I overnight at cell density 2 × 104 per cm^2^. The fully attached cells were 80% confluence the next day, subjected to 5%, 25% elongation cyclic stretching at 1 Hz for 24 h. For the no stretch control, the cells were seeded on PDMS membrane, followed by static culture for 24 h in the same CO_2_ incubator as the cells being stretched.

### 2.3. Cell Alignment Measurement

After stretching, the cells were observed under the microscope (Leica Camera Incorporation, Wetzlar, Germany) with bright field. Images were taken randomly at magnification 200×. Five fields of view were analyzed, with each field of view having above 30 cells. The cell orientation or alignment was analyzed using ImageJ software (National Institute of Health, Maryland, USA) with the angle analysis tool. Cells with angle equals to and greater than 30°, or equals to and less than −30° were assigned as aligned cells. The cells that were between 30° and −30° were assigned as not aligned.

### 2.4. Immunofluorescence Assay

Cells were fixed with 4% paraformaldehyde for 20 min, washed with phosphate-buffered saline PBS, then incubated with Triton X-100 for 10 min (0.5% *v*:*v*). Blocking was done by incubating with 5% (*w*:*v*) bovine serum albumin (BSA) for 30 min, washed with PBS, incubated with primary antibody overnight at 4 °C. After binding to the primary antibodies: FAM38A antibody (Piezo1) (Biorbyt, orb183601, Cambridge, United Kingdom), Desmin antibody [DE-U-10] (GeneTex, GTX26322, Irvine, CA, USA), cells were further incubated 1 h with Alexa Fluor-conjugated anti-rabbit, or anti-mouse (Jackson Immunoresearch, 1:1000, West Grove, PA, USA) with gentle shaking at room temperature. Finally, cells were stained and mounted using the Prolong®Diamond Antifade Mounting Medium containing 4′,6-diamidino-2-phenylindole DAPI (Life Technology, Carlsbad, CA, USA). Cells were analyzed microscopically with an Olympus DP71 device by magnification of 100×, and 200×.

### 2.5. Human Phospho-kinase Array

The phosphorylated proteins were analyzed by human phospho-kinase array (R&D Systems; ARY003B, Minneapolis, MN, USA). The protein lysates were collected, quantified with Bio-rad assay, subjected to the array the following day. Total protein of 100 μg was used to incubate with the membranes containing array antibodies. The Part A and B arrays were blocked in Array Buffer, followed by overnight incubation with protein lysates in separated wells at 4 °C. The target proteins bound to the array antibodies were detected by incubation with biotinylated detection antibody. For visualization, the enhanced chemiluminescence (ECL) (Thermo Fisher Scientific, Waltham, MA, USA) was added, images taken using ImageQuant LAS-4000 (Fujitsu Life Sciences, Tokyo, Japan).

### 2.6. Piezo1 Inhibition

Calcium ion channel inhibitor GsMTx4 (Tocris Bioscience, 4912, Bristol, United Kingdom) was purchased, prepared in ultrapure double distilled water. The GsMTx4 was given to cells at 1 μM, 3 h prior to cell lysate collection, while cells were still under cyclic stretching. 

### 2.7. Calcium Assay

To analyze calcium ion level, the calcium detection assay kit (Abcam, ab102505, Cambridge, MA, USA) was purchased. The supernatant collected was used for analyzing calcium ion release. The cell lysates collected were used for analyzing cytosolic calcium ion level. The assay was performed immediately after the supernatant, or the cell lysates were collected. Measurement was performed in a 96-well plate, at 575 nm absorbance. 

### 2.8. Western Blotting

To collect cell lysates, cells were lysed in ice-cold Radioimmunoprecipitation assay (RIPA) lysis buffer, protein concentration quantified by Bio-rad assay kit (Bio-Rad Laboratories, Hercules, CA, USA). The protein separated by sodium dodecyl sulfate-polyacrylamide gel electrophoresis. After gel electrophoresis, separated proteins were transferred to polyvinylidene fluoride (PVDF) membrane, immunoblotted with primary antibodies prepared in phosphate-buffered saline with 0.05% Tween-20 PBST with 1% BSA overnight at 4 °C with gentle shaking: Anti-Actin Antibody, clone C4 (Merck Millipore, MAB1501, Billerica, MA, USA), lipoprotein receptor-related protein 6 LRP6 (CST, #3395, Beverly, MA, USA), β-catenin (BD, 610154, San Diego, CA, USA), JNK (CST, #9252, Beverly, MA, USA), P-JNK^T183/Y185^ (CST, 9251S, Beverly, MA, USA), GSK3β (Millipore, 07-1413, Billerica, MA, USA), P-GSK3β^S9^ (Millipore, 05-643, Billerica, MA, USA), eNOS (BD, 610297, San Diego, CA, USA), P-eNOS^S1177^ (BD, 612393, San Diego, CA, USA), AKT (Cellsignal, #9276s, Beverly, MA, USA), P-AKT^S473^ (Cellsignal, #3879, Beverly, MA, USA). After primary antibody incubation, membranes with transferred proteins were washed with PBST three times, incubated with secondary antibodies conjugated to horseradish peroxidase, prepared in 5% skim milk for 1 h at room temperature with gentle shaking. Secondary antibodies were discarded, washed with PBST three times. The signals were visualized by exposing to enhanced chemiluminescence using ECL reagents (Thermo Fisher Scientific, 32106, Waltham, MA, USA). The ImageQuant LAS4000 (Fujitsu Life Sciences, Tokyo, Japan) was used for taking images.

### 2.9. Clustered Cells Scoring

The cells formed spheroids, clustered of cells after subjected to 24 h stretching. Bright-field images with magnification 100× were taken, clustered cells were scored from the images. The clustered cells visualized to be 10 cells and above in total with spherical formation were counted. The number of clustered cells was counted in three random fields of view, mean values derived using Excel, graph drawn using GraphPad Prism5 software.

### 2.10. Hydrogel Cultivation of Cells

The cells formed spheroids, clustered of cells after subjected to 24 h stretching. First, PDMS with the cells after stretching was place in a new culture plate. The culture plate was moved under the observation area of microscope to location of the clustered cells. To create a 3D culture system, the clustered cells were picked up using pipette tip attached to a pipetteman by aspiration, transferred to solution of hydrogel (The Well Bioscience Incorporation, Newark, NJ, USA) mixed with complete medium in 1:1, or 1:2 volume ratio. To pick up the clustered cells individually, the lid of plate was opened at an angle below 45 degrees, pipette tip was moved to the location of the clustered cells, picked up by pipette aspiration. After aspiration of the cells, the cells were transferred to the hydrogel and medium mixture in the laminar flow hood. The mixture of hydrogel, medium with cells were further mixed thoroughly before placing in well of 24-well plate. The hydrogel mixed with medium underwent gelation after 15 min incubation in the CO_2_ incubator. Culture media was added to cover the stabilized gel. The hydrogel with cells were cultured for 7 days, cover media changed every 2 days.

## 3. Animal Study

To analyze the potential change in Wnt signaling molecules, a cardiomyopathy animal model was induced. The animal preparation is based on a previous method [13]. Eight-week-old male Wistar-Kyoto (WKY) rats weighing 250–350 g were obtained from the National Science Council Animal Facility (Taipei, Taiwan), housed in the animal center at Kaohsiung Veterans General Hospital (Kaohsiung, Taiwan), and provided with normal rat chow (Purina, St. Louis, MO, USA) and tap water ad libitum. All animal care and procedures were approved by the Research Animal Facility Committee of Kaohsiung Veterans General Hospital. The number of animals kept in each cage was three for the control, and isoproterenol (ISO) group (*n* = 6 for each group). The control group was intraperitoneally injected with vehicle (normal saline with 0.1% ascorbic acid, volume equaled to ISO injection). The ISO-induced rats received intraperitoneal injections of ISO prepared in normal saline with 0.1% ascorbic acid. The histopathology of acute cardiomyopathy and cardiac fibrosis was validated at 2 mg/kg per day for consecutive 5 days. Before subjected to immunohistochemistry assay, the rats had been monitored for 4 weeks through measurement of tail systolic blood pressure, and echocardiography were performed [13].

### 3.1. Immunohistochemistry Assay

To analyze protein expression in tissue, immunohistochemistry (IHC) was performed. The tissue was embedded in paraffin, deparaffinized, followed by antigen retrieval in microwave with double distilled water. The endogenous peroxidase was removed by adding 3% H_2_O_2_. Tissue blocked in 1% BSA, incubated in primary antibody Wnt1 (Abcam, ab15251, Cambridge, MA, USA) at 4 °C overnight. The next day, tissue was incubated in secondary antibody using the post primary block reagent (Leica Biosystems, Richmond, IL, USA) against mouse, or the Novolink polymer (Leica Biosystems, Richmond, IL, USA) against rabbit primary antibodies. The tissue was visualized with 3,3′-Diaminobenzidine DAB solution with DAB substrate, counterstained with hematoxylin. Dehydration was performed in the sequence of 30%, 50%, 75%, 95% two exchanges, 100% two exchanges of ethanol. When the tissues were air-dried, further dehydrated with two exchanges of xylene before mounting. 

### 3.2. Statistical Analysis

All measurements were produced at least three times under independent conditions. The results are shown as mean ± standard error of the mean (SEM). Statistics were analyzed with one-way ANOVA. *, *p* < 0.05 indicates a significant result, **, *p* < 0.01 indicates a very significant result, ***, *p* < 0.05 indicates a highly significant result.

## 4. Results

### 4.1. Cyclic Stretch Induces Cardiomyocyte Realignment and Piezo1 Redistribution

**Figure 1:**
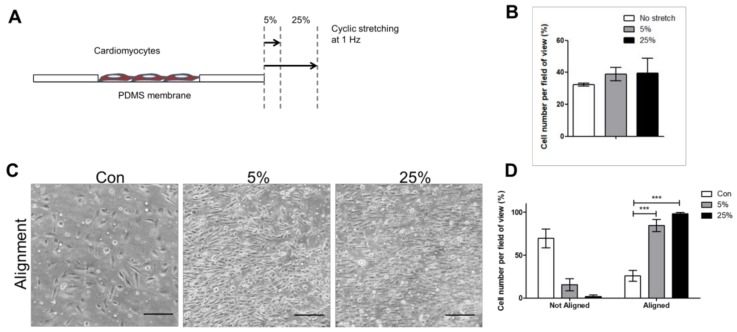

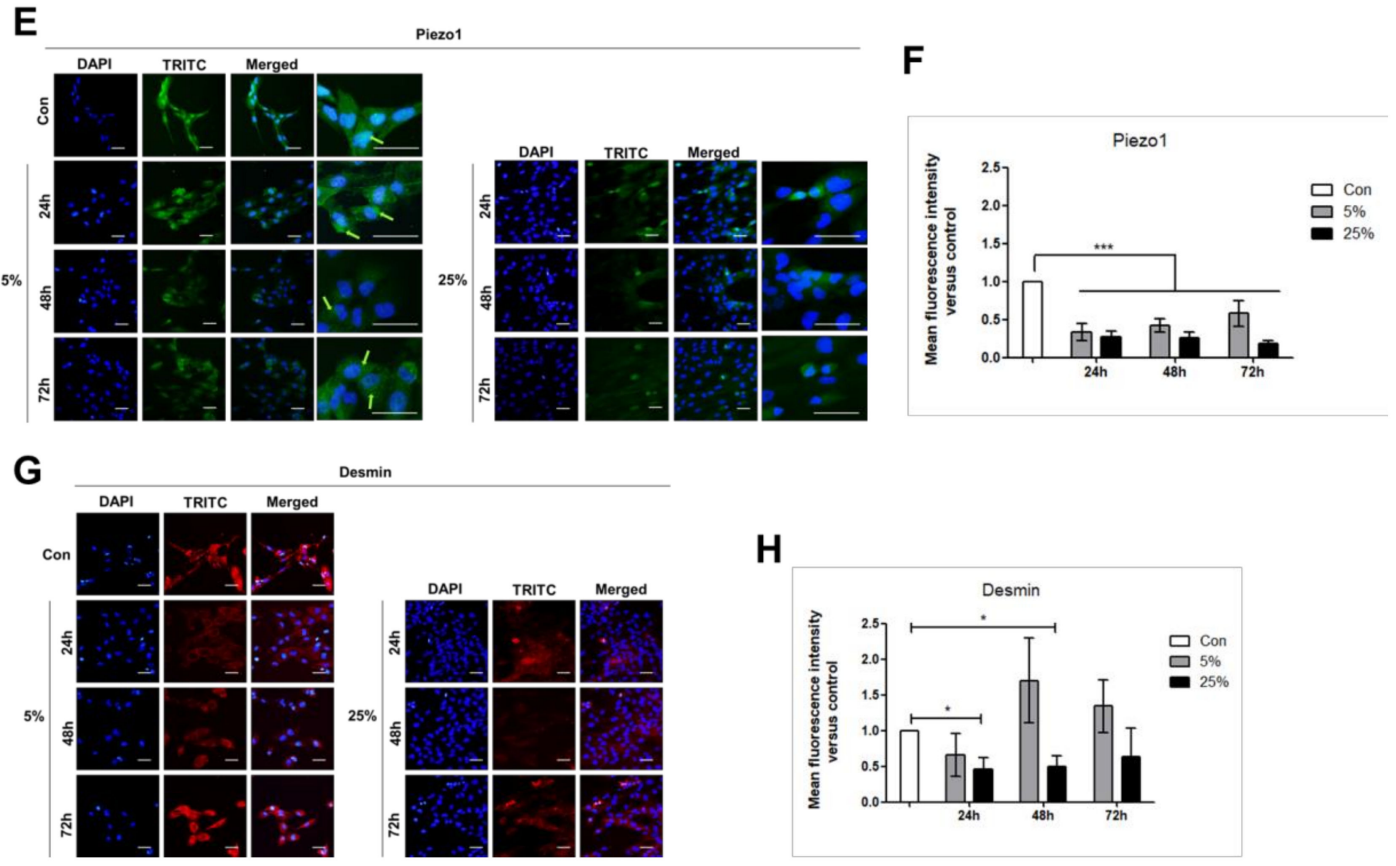
Cyclic stretch affects cardiomyocyte alignment and Piezo1 distribution. (**A**) Illustration showing cardiomyocytes seeded on stretchable polydimethylsiloxane PDMS membrane, stretched in one direction or uniaxial. (**B**) Cell number was counted after subjected to 5%, and 25% 24 h stretching. (**C**,**D**) Cell alignment after 24 h was measured using ImageJ. (**E**,**F**) Expressions of the stretch-activated ion channel Piezo1. (**G**,**H**) and cardiomyocyte characteristic marker Desmin analyzed by immunofluorescence assay after 24 h, 48 h, and 72 h of stretching. Scale bar = 100 μm. *, *p* value < 0.05, ***, *p* value < 0.001.

To test the hypothesis that cardiomyocytes respond to mechanical stimulation, cells were subjected to 5% and 25% cyclic stretching at 1 Hz for 24 h. Results showed no significant change in cell growth (Figure 1A,B); however, cells were aligned to the stretching force under both 5% and 25% (Figure 1C,D). The Piezo1 protein expression decreased under both 5% and 25%. The cardiomyocyte characteristic marker Desmin decreased at 25% compared to 5% after stretching at 24, 48, and 72 h (Figure 1E,H). Since Desmin is a characteristic marker expressed by muscle cells and is expressed in AC16, reduced expression of Desmin implies that the cells were losing myocyte characteristic.

### 4.2. Cyclic Stretch Stimulates the LRP6/β-Catenin Signaling

**Figure 2:**
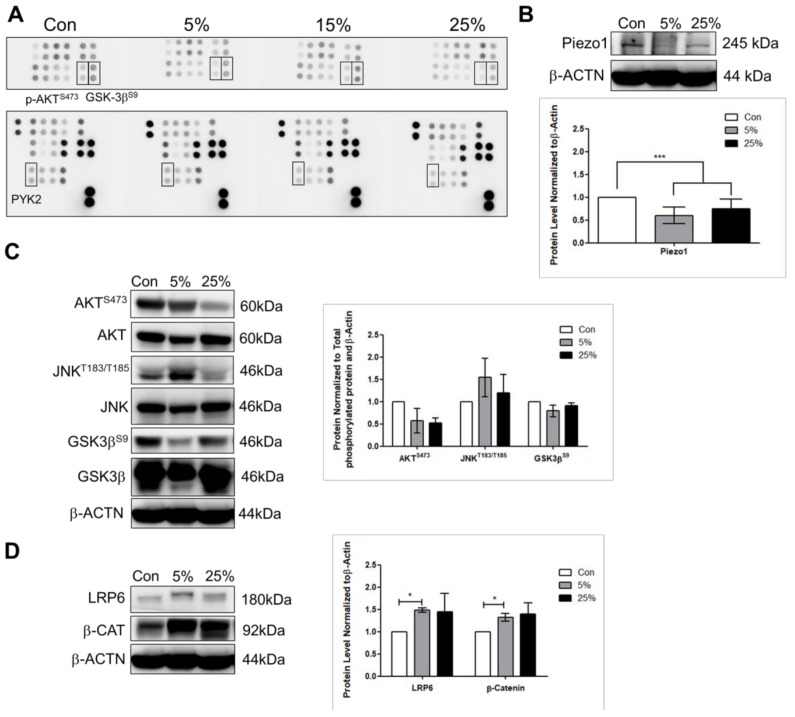
Cyclic stretch stimulates the low-density lipoprotein receptor-related protein 6 LRP6/β-catenin signaling. (**A**) Cardiomyocytes analyzed by Phospho-kinase array at no stretch (control), 5%, 15%, and 25% 24 h of stretching. (**B**) Western blot performed to analyze the stretch-activated ion channel Piezo1 protein expression. Validation of the (**C**) phosphorylated-Ak transforming serine473 (AKT^S473^), phosphorylated-c-Jun N-terminal kinase threonine183/tyrosine185 (JNK^T183/Y185^), phosphorylated-glycogen synthase kinase-3 beta serine9 (GSK3β^S9^), total AKT, JNK, and GSK3β, (**D**) wingless integrated (Wnt) signaling molecules LRP6, and β-catenin. Data represent at least three independent experiments in SEM ± SD. *, *p* value < 0.05, ***, *p* value < 0.001.

**Figure 3:**
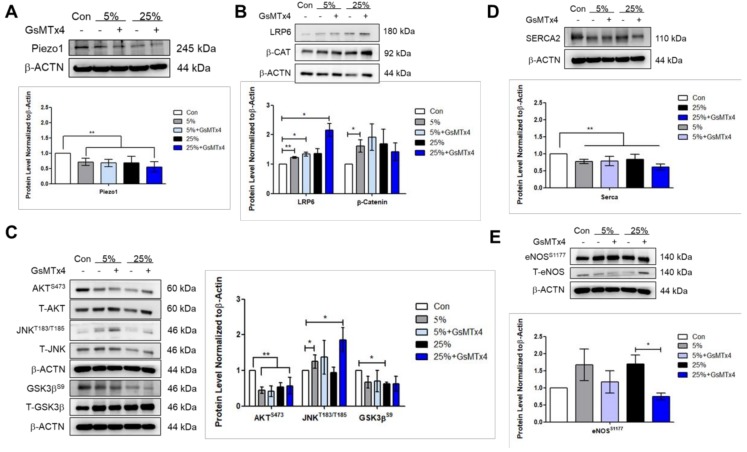
Aggressive mechanical stimulation further activates the LRP6/β-catenin signaling when Piezo1 was inhibited. Western blot showing the protein expressions for (**A**) Piezo1, (**B**) LRP6, and β-catenin, (**C**) phosphorylated AKT^S473^, JNK^T183/Y185^, GSK3β^S9^, and total AKT, JNK, and GSK3β. (**D**,**E**) Protein level for the calcium ion regulator sarco/endoplasmic reticulum Ca^2+^ SERCA2, phosphorylated-endothelial nitric oxide synthase serine1177 P-eNOS^S1177^, and total eNOS. Data represent at least three independent experiments in SEM ± SD. *, *p* value < 0.05, **, *p* value < 0.01.

To examine the effect of mechanical stimulation on cardiac function- associated protein expressions, phospho-kinase array was performed for no stretch (control), 5%, 15%, and 25% elongation for 24 h. These studies showed changes in P-AKT^S473^, P-GSK3β^S9^, and the calcium ion channel protein tyrosine kinase 2 PYK2 expression levels (Figure 2A). Consistent with the immunofluorescence result, the Piezo1 protein level decreased under 5%, and 25% compared to that control (Figure 2B). Furthermore, the P-JNK^T183/Y185^ increased under 5% (Figure 2C); whereas the Wnt signaling molecules LRP6 (low-density lipoprotein receptor-related protein) and β-catenin increased significantly at 5% compared to that control (Figure 2D). To investigate if the Wnt signaling was activated through Piezo1, Piezo inhibitor GsMTx4 was added to cells during stretching. Interestingly, the LRP6 and P-JNK^T183/Y185^ were further increased when Piezo1 was inhibited under 25% (Figure 3B,C). Consistent with the phospho-kinase array result for protein tyrosine kinase 2 PYK2, the protein level of the calcium ion channel, sarco/endoplasmic reticulum Ca^2+^ (SERCA2) was found to be decreased at 5% and 25% (Figure 3D). The mechanical stimulation altered of total eNOS production, but the P-eNOS^S1177^ had no significant change (Figure 3E). However, inhibiting Piezo1 led to significantly lowered P-eNOS^S1177^ under 25% when normalized to T-eNOS and β-actin (Figure 3E).

### 4.3. Aggressive Mechanical Stimulation with Inhibited Piezo1 Reduces eNOS Level

**Figure 4:**
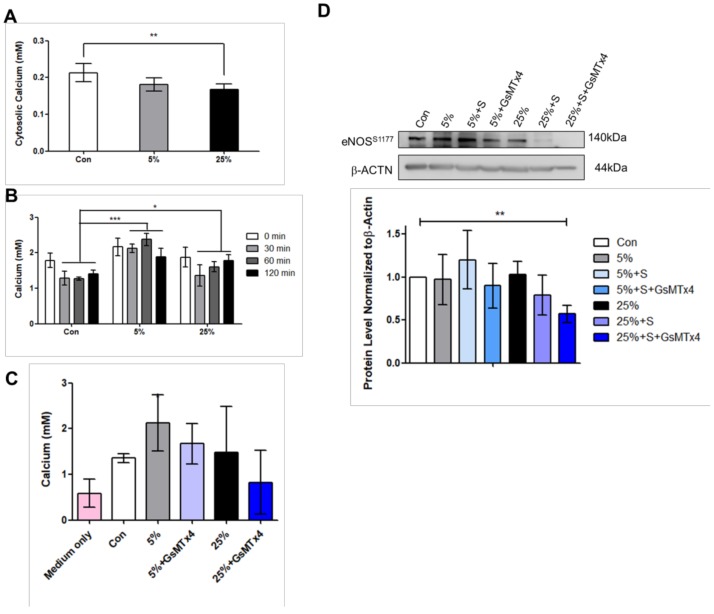
Aggressive mechanical stimulation reduces calcium ion and P-eNOS levels. (**A**) Cardiomyocytes cell pellets were collected and lysed in lysis buffer after 24 h stretching, cytosolic calcium ion determined by colorimetric detection kit. (**B**) Calcium ions released into the medium was measured immediately after stretching at 0 min, 30, 60, and 120 min. The medium which was the supernatant were collected simultaneously, absorbance measured at 575 nm. (**C**) Calcium ion released into the medium was measured after 24 h stretching with Piezo1 inhibited (Piezo1 inhibitor GsMTx4) at 5%, and 25%. (**D**) After 21 h of stretching, the cardiac drug simvastatin, or GsMTx4 was added to the cells, and continued cultured for 3 h before collecting cell lysates at 24 h. Data represent at least three independent experiments in SEM ± SD. *, *p* value < 0.05, **, *p* value < 0.01, ***, *p* value < 0.001.

**Figure 5:**
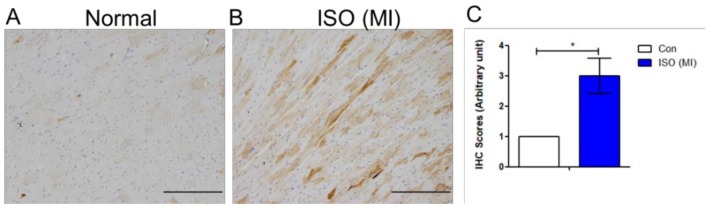
Expression of the Wnt1 protein in the isopretenol-induced myocardial infarction rat model. (**A**) Analysis of Wnt1 protein expression in normal rat by immunohistochemistry. (**B**) Wnt1 protein expression in the isopretenol (ISO)-induced myocardial infarction (MI) rat model (*n* = 3). (**C**) Scoring of the tissue slides analyzed by immunohistochemistry: 0 (no staining); 1 (<10% staining); 2 (10–50% staining); 3 (>50% staining). *, *p* value < 0.05.

During cardiac overloading, the transport of calcium ions level in and out of cells is changed. Consistently, the calcium ion level in cytosol decreased significantly at 25% when compared to control (Figure 4A). Calcium release was higher at 5% compared to control and 25% (Figure 4B); however, no significant change of calcium release when Piezo1 was inhibited (Figure 4C). Furthermore, the potential of the cardiac model was examined using the cardiac drug, simvastatin because it enhances eNOS production to maintain cardiac cell function. The cells were given 10 μM simvastatin. With simvastatin, P-eNOS^S1177^ was slightly increased at 5%, slightly reduced at 25%, and even lower at 25% when Piezo1 was inhibited (Figure 4D). This evidence shows that the clinical drug simvastatin was improving the eNOS protein which helped to maintain cardiac function. Supporting the idea that increased LRP6/β-catenin due to aggressive mechanical stimulation were associated with cardiomyopathy, the result from immunohistochemistry of isoproterenol-(ISO) induced myocardial infarction animal model showed increased Wnt1 expression compared to that control rat (Figure 5A,C).

### 4.4. Cyclic Stretch Enhances Growth of Clustered Cells Expressing Piezo1 that Survived in 3D Hydrogel

**Figure 6:**
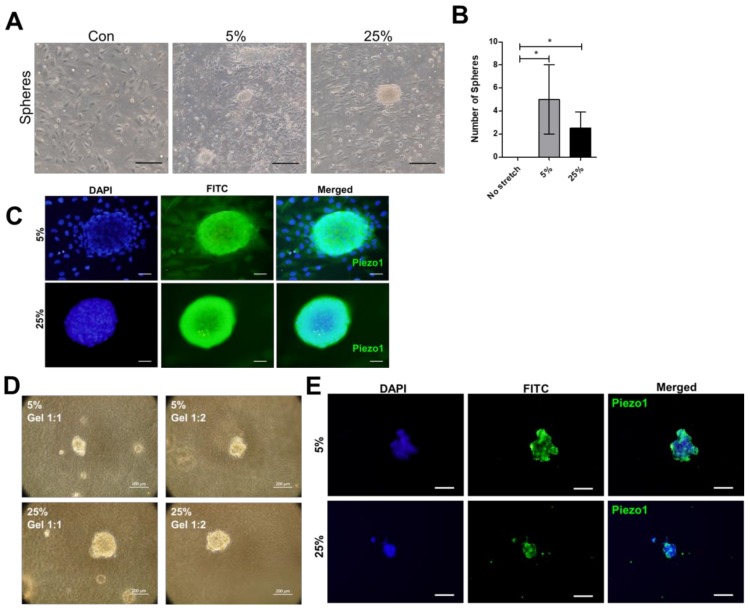
Cyclic stretch enhances growth of clustered cells expressing Piezo1 that survive in 3D hydrogel. (**A**,**B**) The 24 h stretching formed clustered cells with spherical appearance. Scale bar = 100 μm. (**C**) Immunofluorescence assay detected Piezo1 in the clustered cardiomyocytes. Scale bar = 20 μm. (**D**) The clustered cardiomyocytes were transferred to hydrogel, cultured for 7 days with cover medium changed every 2 days. Scale bar = 200 μm. (**E**) After 7 days, the cells in hydrogel were embedded in paraffin, analyzed for Piezo1 expression by immunohistochemistry. Scale bar = 200 μm. Data represent at least three independent experiments in SEM ± SD. *, *p* value < 0.05.

The heart is three dimensional in nature, the potential of cardiomyocytes expressing Piezo1 in 3D culture was investigated. Clustered cardiomyocytes expressing Piezo1 were observed after 24 h stretching (Figure 6A,B). The clustered cells which were spherical in appearance were picked up individually and transferred to hydrogel for culture. After 1 week of culture in two concentrations of hydrogel, the clustered cells survived (Figure 6D). In the hydrogel, the survived clustered cells expressed Piezo1 at 5% and 25% (Figure 6E).

## 5. Discussion

The heart experiences electrical, biochemical, and mechanical stimulations as it beats; thus, cardiac cells express mechanosensors that can detect the mechanical stimulation in nature [2]. We hypothesized that dynamic culture of cardiomyocytes under mild and aggressive mechanical stimulations can affect cardiac cell physiology and pathology, respectively. This cardiac physiology includes the expression of cardiomyocyte marker Desmin [14], and facilitation of calcium ion transport through the sarco/endoplasmic reticulum Ca^2+^-ATPase (SERCA2) for contractility [15,16]. The cardiac pathology includes change in total eNOS production which maintains cardiac function [17,18]. According to Lewis A. H. et al., Piezo1 responses to repetitive stimulation in frequency-dependent manner in which mechanical stimulation with higher frequency can cause higher probability of Piezo1 channel opening [19]. It has been shown that Piezo1 is required for structural maintenance of endothelial cells, and the remodeling of vascular smooth muscle cells during hypertension [20]. Study on the role of Piezo1 in cardiomyocytes is lacking; however, mechanical stretch was shown to increase cardiac hypertrophy in mice that expressed the stretch-responsive G-protein-coupled receptor, APJ [21]. We observed that mild stretch at 5% resulted in higher Desmin expression (Figure 1G,H), higher level of calcium ion released (Figure 4B,4C), as well as higher P-eNOS production compared to aggressive stretch at 25% (Figure 4D). In addition, the cytosolic calcium ion was found to be lower in the 25% compared to 5% (Figure 4A). Previous study revealed that Piezo1 is up-regulated by Angiotensin II in a heart failure rat model [22]. Our observation that Piezo1 is decreased compared to control in the 5% and 25% (Figure 2B) stretch suggests that the stretched cardiomyocytes were not that of heart failure condition, and Piezo1 decrease may be acting to counteract cardiomyopathy.

Previous study has shown that the JNK signaling is activated in the heart with contractile dysfunction [23], and the Wnt signaling is activated in the heart disease model [24]. Furthermore, the JNK increase has been shown to be related to cardiomyocyte fibrosis and apoptosis during cardiac pressure overload [25]. As a result, the further activation of JNK signaling under 25% with inhibited Piezo1 indicates that the cardiomyocytes might be under mechanical overloading (Figure 3C). Consistent with Pahnke A. et al. review in which the Wnt activation is reported to be involved in cardiac development, repair, and disease [26]. Mechanistically, the Wnt binds to receptor Frizzled (Frz), as well as low-density lipoprotein receptor-related protein 5 or 6 (LRP5/6) to stabilize cytosolic β-catenin through glycogen synthase kinase 3 (GSK-3) inhibition. Consistent with this idea, we observed that total protein level of the Wnt signaling molecules: LRP6 and β-catenin increased when cardiomyocytes were subjected to mild and aggressive stretching (Figure 2C,D and Figure 3B,C). 

Our experiments demonstrated that cardiomyocytes response to mild and aggressive mechanical stimulations. Both LRP6 and JNK are increased at 5% and 25% stimulations; however, were further increased at 25% when Piezo1 was inhibited. The Wnt signaling regulates cardiac cell metabolism such as genes related to arteriosclerosis [27]. We proposed that this further increase or reactivation effect at 25% attempts to rescue P-eNOS production to maintain cardiac function during cardiac overloading. In regard to Piezo1 inhibition at 25%, the decrease in calcium ion release may have affected the P-eNOS production. As a result, the cardiac-protective drug, simvastatin slightly increased P-eNOS^S1177^ at 5%; however, P-eNOS^S1177^ was reduced at 25% (Figure 4D).

**Figure 7:**
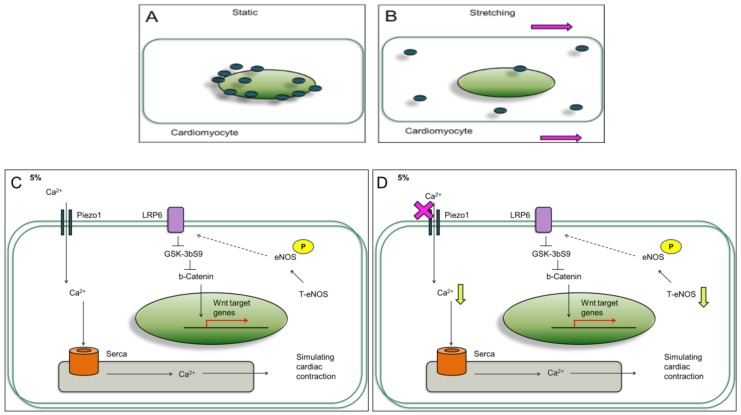

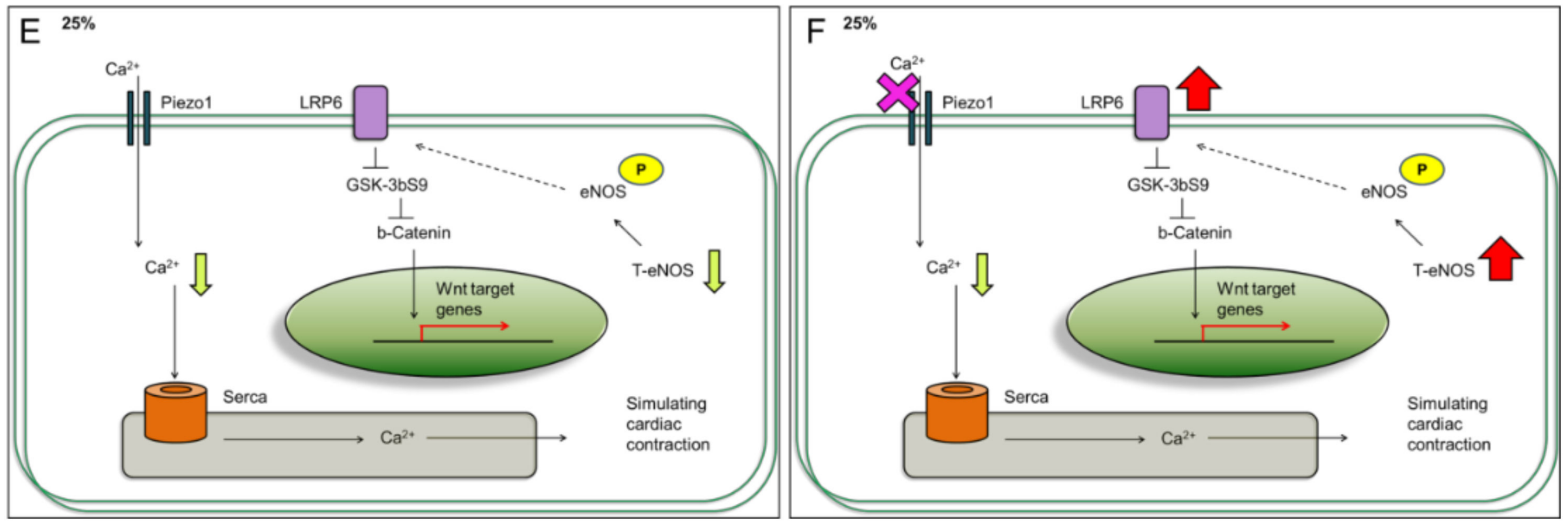
The effects of mild and aggressive mechanical stimulations on LRP6, JNK signaling, eNOS production, and calcium ions in cardiomyocytes. (**A**,**B**) Piezo1 distribution observed to be different after subjected to mild and aggressive mechanical stimulations. (**C**) Cardiomyocytes subjected to 5% stretching for 24 h increased the LRP6/β-catenin (Wnt pathway) signaling. The SERCA2 is known to act as calcium transporter on the sarco/endoplasmic reticulum to promote cell contractility. In this study, SERCA was detected in the cardiomyocytes. (**D**) Piezo1 inhibition reduced calcium release at 5%. (**E**) At 25%, calcium ion release and T-eNOS protein level were reduced compared to 5%. (**F**) Piezo1 inhibition at 25% reduced calcium ion release; however, significantly increased LRP6/β-catenin, and JNK.

In contrast, when Piezo1 was inhibited under 5% mild stretching, the LRP6 and JNK further activation effect was not observed (Figure 3B,C). This suggests that the mechanical stimulation magnitude or loading experienced by the cardiomyocytes was different at 5% compared to 25%. Our study is consistent with Volkers L et. al. study in which the stretch-activated ion channel (SAC) is able to sense mechanical loading [28]; therefore, the cardiomyocytes respond differently to mild and aggressive stimulations. The increased LRP6 and JNK signaling observed through cyclic stretching may have involved Piezo1. We observed that (1) 5% induced LRP6 and JNK signaling (Figure 2C,D and Figure 3B,C) with enhanced calcium ion release (Figure 4B); (2) 25% induced LRP6 and JNK signaling (Figure 2C,D and Figure 3B,C) with reduced calcium ion release at the time points 0, 30, 60, 120 min (Figure 4B). These results imply that cardiomyocytes experiencing higher loading under 25% have imbalance calcium ions maintenance. A previous review implied that blood volume can have two kinds of impact on the cardiac cells: (1) stressed; (2) unstressed [29]. Here, the cardiomyocyte dynamic models may be associated with a physiological beating heart rate (cells stretched at 1 Hz) with different mechanical stimulation strengths: 5%, and 25%. The cardiomyocytes experiencing mild mechanical stimulation express LRP6 and JNK, and no significant change observed when Piezo1 was inhibited (Figure 7C,D). On the contrary, cardiomyocytes experiencing aggressive mechanical stimulation express LRP6 and JNK, and were further increased when Piezo1 was inhibited (Figure 7E,F). Since Piezo1 has been reported to be activated by stretching, the effects observed were most likely linked to Piezo1. Surprisingly, the level of Piezo1 decreased when cardiomyocytes were experiencing the different strengths of mechanical stimulations. Not only the Piezo1 level decreased compared to static or control, but the Piezo1 distribution throughout the cardiomyocytes was observed to be different (Figure 1E,F and Figure 7A,B). Perhaps, the higher Piezo1 level in the static culture was due to lack of mechanical stimulations, or lack of dynamics. In nature, the heart does not remain or survive in static environment. In consequence, the higher Piezo1 level observed in the static culture condition may imply a diseased condition which requires further investigation.

Our previous results showed that increased blood pressure activates Wnt signaling via down-regulation of GSK3β-mediated pathway in the central nervous system [30]. Furthermore, it was reported that hypertension disrupted heart rate, sudden increase in heart rate potentially lead to heart failure [31]. Our efforts to replicate cardiomyocytes response to different mechanical stimulation stresses show that cardiomyocytes express mechanosensor Piezo1 which plays a role in regulating calcium level and P-eNOS protein level. 

Our result demonstrates a connection between mechanosensor Piezo1 and the impact of mechanical stimulations on cardiomyocytes. The increased LRP6, JNK, P-eNOS and calcium ion release at 5% compared to 25% indicate that cardiomyocytes respond to the mild and aggressive mechanical stimulations differently. This study provides unique evidence which shows that mechanical stimulation can influence proteins associated with cardiac function through Piezo1, a factor that is important in the naturally dynamic microenvironment.

## 6. Conclusions

Mechanical force potentially sensed by Piezo1 involves the LRP6/β-catenin signaling, and JNK signaling. Piezo1 inhibition did not alter the LRP6/β-catenin and JNK signaling pathways under 5% stretching. However, both LRP6/β-catenin and JNK signaling pathways were further activated under 25% stretching when Piezo1 was inhibited. It is concluded that the further activated LRP6/β-catenin and JNK signaling were a result of higher stress under 25%.
